# The roles of community health workers in management of non-communicable diseases in an urban township

**DOI:** 10.4102/phcfm.v6i1.693

**Published:** 2014-11-21

**Authors:** Lungiswa P. Tsolekile, Thandi Puoane, Helen Schneider, Naomi S. Levitt, Krisela Steyn

**Affiliations:** 1 School of Public Health, University of the Western Cape, South Africa; 2 Faculty of Health Sciences, Department of Medicine, University of Cape Town, South Africa

## Abstract

**Background:**

Community health workers (CHWs) are increasingly being recognised as a crucial part of the health workforce in South Africa and other parts of the world. CHWs have taken on a variety of roles, including community empowerment, provision of services and linking communities with health facilities. Their roles are better understood in the areas of maternal and child health and infectious diseases (HIV infection, malaria and tuberculosis).

**Aim:**

This study seeks to explore the current roles of CHWs working with non-communicable diseases (NCDs).

**Setting:**

The study was conducted in an urban township in Cape Town, South Africa.

**Method:**

A qualitative naturalistic research design utilising observations and in-depth interviews with CHWs and their supervisors working in Khayelitsha was used.

**Results:**

CHWs have multiple roles in the care of NCDs. They act as health educators, advisors, rehabilitation workers and support group facilitators. They further screen for complications of illness and assist community members to navigate the health system. These roles are shaped both by expectations of the health system and in response to community needs.

**Conclusion:**

This study indicates the complexities of the roles of CHWs working with NCDs. Understanding the actual roles of CHWs provides insights into not only the competencies required to enable them to fulfil their daily functions, but also the type of training required to fill the present gaps.

## Abstract (fr)

**Le rôle des agents de santé communautaires dans la gestion des maladies non-contagieuses dans un township urbain.**

**Contexte:**

Les agents de santé communautaires (CHW) sont de plus en plus considérés comme un élément capital du personnel de santé en Afrique du Sud et dans d’autres parties du monde. Les CHW assument divers rôles, tels que l’autonomisation des communautés, la prestation de services et le lien entre les communautés et les services de santé. Leurs rôles sont mieux compris dans les domaines de la santé maternelle et infantile et les maladies infectieuses (infection par le VIH, le paludisme et la tuberculose).

**Objectif:**

Cette étude examine les rôles actuels des CHW s’occupant des maladies non contagieuses (NCD).

**Cadre:**

L’étude a été menée dans un township urbain du Cap, en Afrique du Sud.

**Méthode:**

On s’est servi d’une méthode de recherche qualitative et naturaliste qui utilise des observations et des entretiens approfondis avec les CHW et leurs superviseurs travaillant à Khayelitsha.

**Résultats:**

Les **C**HW ont des rôles multiples pour soigner les NCD. Ils sont éducateurs sanitaires, conseillers, personnel de rééducation et animateurs de groupes de soutien. Ils décèlent les complications de la maladie et aident les membres de la communauté à mieux naviguer dans le système de santé. Ces rôles sont déterminés selon les attentes du système de santé et les réponses aux besoins de la communauté.

**Conclusion:**

Cette étude indique les complexités des rôles des CHW travaillant avec les NCD. La compréhension des rôles des CHW donne une idée non seulement des compétences requises pour leur permettre de s’acquitter de leurs tâches quotidiennes, mais aussi du type de formation requis pour combler les lacunes actuelles.

## Introduction

Community health workers (CHWs) are increasingly being recognised as a crucial part of the health workforce.^1^ In South Africa and worldwide, CHWs have provided health care to communities for many decades and have assumed a variety of roles, including community empowerment, provision of services and linking communities with health facilities. Their roles are better understood in the areas of maternal and child health.^2^ For the purposes of this article CHWs are defined as health workers with informal job-related training and no professional or paraprofessional tertiary training, and limited in-service training to contribute to patient management at community level. They may receive a stipend or work voluntarily^3^ and work in communities in which they reside.

CHWs’ tasks over the years have evolved from mainly focusing on prevention and promotion to more supportive roles that are associated with the increased burden of chronic lifelong conditions. South Africa, like many countries in transition, has experienced an increase in the burden of chronic lifelong conditions associated with the HIV infection epidemic and the parallel emergence of non-communicable diseases (NCDs). This burden of disease has led to increased workloads, overcrowding at health facilities and poor quality of care,^4^ and has exerted a tremendous strain on human resources in the healthcare system, especially those working at primary healthcare (PHC) level.^5^ The use of CHWs as part of a solution to the human resources crisis in health settings has been suggested.^6,7^

The South African Government has made significant strides in providing free PHC for all, meaning that people with chronic diseases such as NCDs are provided with long-term treatment at no cost and will further need ongoing support to assist them to adhere to treatment. As part of disease management, patients diagnosed with NCDs and placed on treatment are typically advised to attend support groups run or facilitated by professional nurses at the health facilities.^6^ Despite the efforts made at health facilities in managing NCDs, these conditions continue to be managed poorly^8^ and a need for extended and continued care at community level therefore exists.

Given the fact that not all patients utilise health facilities for routine check-ups and that NCDs’ symptoms are not recognised until the late stages when complications are apparent, employing CHWs to increase awareness about prevention and control of diseases in communities is crucial. CHWs could strengthen the link between health services and the community, increasing access to services, especially in underserved communities.^9^

In South Africa CHWs have been largely utilised in programmes that target infectious conditions such as HIV/AIDS and tuberculosis (TB) as well as maternal and child health. Their roles in such programmes are clearly defined and have an established base of evidence illustrating the benefits.^2^ The focus of programmes has been propelled by the need to achieve the Millennium Development Goals, resulting in less attention being given to other conditions such as NCDs.^10^

Numerous interventions for NCDs have been described that utilise the CHW model in disadvantaged communities.^11,12^ Studies from developed countries highlight varied roles and relevance or importance of CHWs in the management of NCDs. These roles include patient education,^13,14^ care and support,^13,15,16^ provision of social support and acting as a liaison with the healthcare system.^3^ For example, in a diabetes intervention CHWs assisted in monitoring blood glucose, blood pressure and potential complications, and provided social support to patients as well as their families.^15^ Despite general support of the CHW model in the management of diseases, there is a need to further understand the roles of CHWs in the prevention and management of NCDs, especially in resource-poor settings.

Recent policy reforms in South Africa prioritise the revitalisation of PHC and stronger community-based services.^17^ On the eve of the re-engineering of PHC and the implementation of PHC outreach teams in South Africa it becomes imperative to understand the roles of CHWs, particularly in caring for conditions previously not included in their work. According to the policy statements on re-engineering of PHC, the work of CHWs has been conceptualised to be comprehensive and to cover household and community level.^18^ With respect to NCDs, at household level their roles will include health promotion, that is education on diet, exercise and lifestyle, and screening for those at high overall cardiovascular disease risk and in particular for diabetes and hypertension, foot care and an integrated approach to adherence support. At community level the focus will be on campaigns, support groups and promoting action on risk factors related to diet and exercise.

### Objectives

In the light of these broadly stipulated roles of CHWs, this study therefore explored CHWs’ current roles in NCDs care with a view to identifying existing challenges. The information gathered will assist in informing a training curriculum that relates to CHWs’ daily activities and can support a meaningful response to NCDs.

### Contribution to field

Understanding the CHWs’ current roles, especially on the eve of re-engineering of PHC, is important for adapting current roles to what will be expected in their new roles in PHC outreach teams. This is especially true as re-engineering of PHC may require an expansion of skills as well as new knowledge. Providing an understanding about current roles will give insight into how CHWs organise their work, the type of activities related to NCDs that they engage in, as well as coping strategies employed in order to address deficiencies in their own work. The results will provide information about the key challenges of managing NCDs at community level, and also highlight possible models that could be used when training opportunities are limited.

## Research methods and design

### Study design

A qualitative naturalistic research design utilising observations and in-depth and unstructured interviews to investigate the actual daily activities of CHWs working with NCD patients was adopted. A naturalistic observation assumes that the roles of CHWs are socially organised and involve examining^18^ the CHWs in their natural environment whilst carrying out their everyday tasks as they would normally do.^19^ The use of naturalistic observations facilitated an understanding of the complex realities of CHWs working in resource-limited settings with clients with NCDs as well as various influences affecting their work.

### Setting

The non-governmental organisation (NGO) studied was located in Khayelitsha, an urban township in Cape Town, South Africa. Khayelitsha is an economically disadvantaged community with predominantly informal dwellings. According to Census data, Khayelitsha subdistrict has an estimated population of 406 779;^20^ however, this number is expected to have increased to over half a million because of an increased influx of people from rural to urban areas over the past few years.^21^ More than half of the population (67%) is unemployed.^22^

The CHWs who work in this area are employed by an NGO operating in Khayelitsha and receiving funding from the Government. Some of the CHWs within the NGO are also involved in TB programmes, where they act as directly observed TB therapy (short course) supporters. In addition, CHWs also work as home-based carers. At the time of the study there were no ward-based teams operating in the area as is the case in other parts of South Africa.

CHWs within this NGO are supervised by professional nurses and coordinators who oversee their daily activities, including administration. However, the ratio of CHWs to supervisors varies amongst organisations. The daily clinic nurse workload in the subdistrict is above the national average, as each nurse provides community-based care for about 33 people per day.^23^ Such findings highlight the importance of community-based services as well as the need for CHWs. Compared to other subdistricts in the Western Cape, Khayelitsha has the highest burden of HIV/AIDS, chronic NCDs and injuries.^24^ This makes it an appropriate setting for encouraging preventive and promotive community health services.

### Study population and sampling strategy

Community health workers employed by the NGO were included in the study. Purposive sampling was used in the selection of one NGO, which was chosen on the basis of location as well as its involvement in the management of clients with NCDs. This NGO had 126 employees at the time of the study.

### Data collection

A total of 10 CHWs were observed. Data were collected during August 2011 over a period of four weeks through observations and unstructured interviews. During observations which occurred during a working day (4.5 hours), the researcher collected data through note taking. Unstructured interviews with CHWs were conducted to stimulate discussions related to roles, issues pertaining to training and understanding of the relations and links with the health facility. These were introduced at the end of each observation when there were questions that stemmed from the observations. Data collected from informal conversations during observation were recorded in the form of notes. This was complemented by the researcher further reflecting on and summarising the proceedings of the day. In order for the researcher to have a clearer understanding of some of the CHW activities and practices observed in the field, in-depth interviews were conducted with two coordinators who supervise CHWs’ daily activities.

In preparation for data collection the researcher spent a week with the CHWs, accompanying them in whatever activities they were carrying out. This served three purposes, namely to familiarise the researcher with the day-to-day activities of the NGO, to gain trust and to establish rapport with the CHWs. In addition, the researcher participated in some of the activities undertaken by the CHWs, such as morning prayers and debriefing sessions. Building trust was an important element in the data collection process and assisted in gaining insider status.

### Data analysis

Data from field notes obtained from observations and in-depth interviews were analysed through thematic content analysis. The data analysis process started with identification of units of meaning, which were then categorised. Central themes were identified after the data were categorised.

### Ethical considerations

Permission to conduct the study was obtained from the Research and Ethics Committee at the University of the Western Cape, the Department of Health and the NGO which employed the CHWs. Voluntary informed consent was requested from the CHWs in order for them to participate in the study.

## Results

Analysis of the field notes revealed several primary themes. Principally, the CHWs had multiple roles and these could be summarised into six broad themes: advisor; provision of direct services; monitoring of clients; linking clients with the health system; capacity building; and administration.

The scenario shown in Box 1 indicates some of the roles and realities of CHWs working in the area of NCDs.

**BOX 1:** Observation of community health workers’ conducting their daily work.

**Scenario**
The day started at the NGO base and I was assigned to accompany a pair of CHWs. We walked for about 20 minutes before reaching a newly formed support group in a more formal settlement. The group was organised and had a chairperson and a secretary who took minutes. The support group opened with a prayer and CHWs commenced with the support group activities. I was introduced to the members as a colleague visiting the club to see the work that CHWs do.
One of the CHWs started taking out the instruments that were going to be used to measure blood pressure, weight and blood glucose. There were about 15 members on this day and I watched them getting ready to be measured. The majority of the members were women and many of them were overweight. A bathroom scale was used to measure weights and an automated blood pressure monitoring machine was used to measure blood pressure.
One CHW conducted weight and blood pressure measurement, whilst the other was responsible for measuring blood glucose. The same cuff size was used on all members. The cuff could not fit one of the women's arms and it was put around the wrist and a measurement was recorded. All the measurements were recorded in a book. Members whose weights were above the scale's threshold could not be measured. One of the members had an elevated blood pressure reading and another one had an above–normal glucose reading. The client with an elevated blood pressure reading was instructed to relax whilst the one with a high blood glucose reading was given water; both clients sat for a about 30 minutes before a second reading was taken. The CHW then wrote a note in her booklet and told both clients that the supervisor, a nurse, would come to observe them the next day.
After measurements were completed an education session was delivered by one CHW who spoke about diabetes and hypertension. The lecture focused on explaining what the conditions are and how they can be prevented, and finished with a lecture on diet. This lecture took about 20 minutes.
Lastly an exercise session was conducted for about 15 minutes. All members of the group participated, including the male participant with elevated blood pressure. After the exercise session CHWs recorded dates for collecting medication from the clinic cards. Thereafter we left the support group.
We walked to another area and found that the support group we were visiting did not convene on that day, as there was a death in the area and all the members went to support the bereaved family. We then proceeded to several households where we delivered medication packets to clients. Once at the household the person who received the medication had to sign in the CHWs’ booklet. Once the CHWs were done we then proceeded to the office to deliver the equipment that was used. This is how the day ended.

NGO, non-governmental organisation; CHW, community health workers

### Advisor

CHWs offered advice to clients, which ranged from health advice to social issues that concerned participants. The following field notes recorded during a support group session demonstrate CHWs’ advisory role which extends beyond the health domain:

After the health education session at the support group, the CHW opened the session for questions. One of the elderly participants enquired about the registration process for the Child Support Grant. The CHW could not offer concrete advice but promised to ask relevant people that may have appropriate answers. (Field notes, support group, day 2)

The advisory role was also confirmed by the coordinators during an in-depth interview and one commented as follows: ‘Community health workers are there to advise clients on number of issues such as what they should eat and where to go in order to get help’ (Co-ordinator, day 8).

### Provision of direct services

It was evident from the observations that CHWs provided direct services to their clients which included facilitation of support groups, health education, distribution of medication as well as rehabilitation exercises.

#### Facilitation of support groups

Facilitation of support groups consisting of clients with diabetes and/or hypertension was one of the roles identified. As facilitators, CHWs assumed a leadership position:

On arrival at the support group the participants were already sitting and waiting for the CHW. She introduced herself and then explained how the session will be structured. Thereafter she started with the day's activities. (Field notes, support group, day 1)

This was also observed in three other support groups that were facilitated by CHWs, where CHWs led support group sessions.

#### Health education

At the support groups and within households, CHWs work as educators. Education sessions offered by CHWs varied and included matters pertaining to nutrition in the management of NCDs as well as explaining about risk factors, symptoms and prevention measures for diabetes and hypertension. In certain instances CHWs explained healthy eating as consuming plenty of fruit and vegetables. The observation highlights gaps in knowledge on diet and nutrition:

I attended an education session delivered by a CHW at a support group. She started the session by describing hypertension; she further explained the risk factors for hypertension and how conditions such as hypertension can be prevented. She completed the session with a lecture on nutrition and how nutrition can prevent diabetes and hypertension. The CHW emphasised the importance of a ‘healthy diet’ and explained that this diet should consist of vegetables and fruit. The information provided to the participants was in the form of a didactic lecture with no illustrations or educational materials to assist the participants in their learning and the CHW in her teaching. (Field notes, support group, day 3)

In addition, health education also occurred during home visits; however, the health education at homes was not of the same depth as within support groups. Health education provided during home visits was in response to questions posed by a particular client rather than routine practice as done in support groups.

#### Distribution of medication

Chronic medication for selected clients assigned to CHWs was distributed in households and at the designated support groups. The delivery of medication to clients by CHWs is a way of improving access to treatment and serves a dual purpose for both the clients and the health facility, by not only ensuring that clients receive medication timeously, but also enabling them to bypass the long queues and long waiting times usually encountered at health facilities and the transport costs involved:

After recording the medication in the record book, we went to deliver the medication to clients. We first visited a client that was bedridden to deliver her medication; we then visited a support group where we delivered the bulk of the medication. All the clients collected their medication and were then reminded about their next doctor's appointment. (Field notes, support group, day 6)

In certain instances CHWs did emphasise the importance of taking medication as prescribed; however, this was not a common practice and was mostly communicated during household visits.

Although education about medication was not included in the sessions with clients, CHWs still assisted with medication-related issues and communicated these to the health facility or nurse supervisor. This is highlighted in the scenario and conversation about what CHWs do when confronted with issues relating to medication:

When CHWs delivered medication at a client's house, the client could not recognise some of the medication that was included in the package and relayed his concern to the CHW. (Field notes, home visit, day 8)

The CHW resolved this issue through a verification process:

‘I look at the boxes, write down the substances, the milligrams [*dosage*] and the drug name that is written in the box. I then go to the book where we record the medication to see if what is given corresponds with what is in the book. If I’m not sure, I ask the nurse supervisor or pharmacist at the clinic. After getting a response I then tell the client what I have been told.’ (Conversation with a CHW, support group, day 6)

#### Rehabilitation services

A single CHW was observed providing exercises to a client with hemiplegia resulting from a stroke. The CHW was assisted by another CHW to handle the client. Whilst conducting the exercises the CHW kept communicating with the client, who seemed pleased with the service provided. The CHW then encouraged the client to visit the local health facility in order to access further rehabilitation services. Interestingly, no clients from the other support groups were observed receiving rehabilitation exercises. The lack of CHWs assisting clients with rehabilitation exercises in other support groups was explained by the co-ordinator:

‘A few years ago we received funding to train CHWs to work with community members that could not readily access physiotherapy services. Our CHWs were then trained in rehabilitation exercise; however, when the funding ended we could not continue with the work we were doing. Currently we only have two CHWs who were part of that training and others have retired.’ (Co-ordinator, day 7)

### Monitoring of clients

CHWs assisted support group members with information that would enable them to better manage their conditions. Whilst conducting support groups, the CHWs collected anthropometric measurements, namely blood pressure, blood glucose and weights of all the participants. Blood pressure was measured using only one cuff size, despite varying mid-upper arm circumferences. Notably, in cases where the cuff was too small to fit the size of upper arm, CHWs placed the cuff on a client's wrist. Finger-prick blood glucose was measured using a new lancet for each person, and clients’ readings were recorded. Weights were measured using a dial bathroom scale and put on surfaces that were sometimes uneven. During weight measurements at no point was the scale calibrated. Calculations of body mass index were not performed. In all the observations, individuals with abnormal blood pressure and/or blood glucose readings were informed of their results and referred to the facility. The appreciation on the faces of the support group members was clearly evident during the taking of measurements. During home visits no CHWs were observed taking any client measurements.

### Linking clients with the health system

At the support groups CHWs referred people who were identified with elevated blood glucose levels as well as raised blood pressure to the nurse supervisor, who then referred the clients to the nearest health facilities. Referred clients were given a referral letter by the nurse to present to the health facility. Although clients reported being attended to at the health facility, no letters were sent back to the nurse supervisor or CHW. When clients were followed up by CHWs through home visits, CHWs could only rely on the clients’ recollection of their clinic attendance and by inspecting the clinic card to ensure that the client did indeed visit the health facility as instructed.

### Capacity building

#### Peer educator

CHWs assisted other CHWs with ‘on-the-job’ experience. It was evident from one of the field observations that CHWs also acted as peer educators by assisting peers with the skills required to fulfil daily tasks:

On one of the visits I noticed that CHWs were working as a trio and one CHW would constantly be observing what was happening. When I enquired about this, I was told that the third CHW was newly recruited and therefore did not have experience in doing the work, thus they had to teach her what is usually done. (Field notes, support group, day 9)

According to the coordinators, this was a way of infusing knowledge about field work to new recruits and to assist them in gaining on-the-job experience. One of the coordinators commented as follows:

‘We have team leaders that we use in the field; they are the ones that we use to partner with a newly recruited CHW. […] the team leaders are CHWs who have been working for the organisation for a long time and they really know the work.’ (Co-ordinator, day 7)

### Administration

CHWs completed forms with information relating to the clients on a daily basis. The information recorded includes patients’ medication, and particulars of the clients to be visited on that day. In addition, CHWs completed daily statistic sheets for clients (clients seen the day before or planned to be seen). The completion of forms was a daily activity done at the beginning of the day, whilst some of the forms that involved updating patient information were completed after home visits and submitted to the coordinators on the next day. CHWs were also responsible for collected signatures of clients when they receive medication, so as to keep a record of the clients who have collected their medication.

## Discussion

The findings of this study show that CHWs fulfil a variety of roles in the management of NCDs. As such they have a crucial role in community level NCD public health care in South Africa. It is evident that the varied roles fulfilled by CHWs seem to be in response to clients’ needs. Consequently a degree of flexibility in the way they approach their work may be necessary so as to allow them to react to the community's needs.

In a society where community needs vary, acting as an advisor may be one of the crucial roles played by CHWs. However, as demonstrated in this study, their knowledge and available support material may need to extend beyond health-related issues, especially in communities with social problems. Other studies have shown that CHWs trained in health-related issues such as diabetes management were often confronted with issues unrelated to diabetes, and therefore suggested the need to prepare them for unanticipated non-diabetes issues.^11^ Such expectations pose a challenge to CHWs’ scope of practice and what can be realistically expected of them. In addition, this illustrates the need to form meaningful networks with other community structures such as social development. These findings further support the broader debate that CHWs need to be integrated into Government programmes and be incorporated into professional teams^1^ who can offer support. Furthermore, communication with other team members whilst in the field can provide more streamlined services for the clients.

Playing the role of a health educator meant that CHWs are expected to act as sources of information, creating an expectation that CHWs possess current and relevant information related to NCDs. The study also highlighted the shortfalls in CHWs’ knowledge, in that information provided was not always accurate. Inability to clarify certain nutrition concepts emphasises the need for training and continuous education that is tailored to the context that addresses the everyday realities of CHWs, as well as the need for teaching aids to further enforce and standardise the health information given to clients. Indeed it has been suggested that the community's perception of CHW knowledge, skills and ability to assist communities with their health needs is crucial in inspiring respect and acceptance of CHW services.^9^

As lay workers based in communities, CHWs play a fundamental role in community-based support groups, and as demonstrated in this study, within such groups their roles could vary. As facilitators, CHWs require a diverse range of skills which includes communication skills, as part of facilitation includes communicating with the audience. CHWs set the subject or topics for discussion and have the potential to influence the focus of the group, and as such require leadership skills. This leadership role presents its own challenges and expectations, as it would imply that CHWs are expected to know how to guide and support group members. In addition, increased demands to support and provide information may rise to a point where expectations on the leader may exceed their capabilities.^25^ Thus it is necessary to structure facilitation of support groups in such a way that forges partnerships, and co-ownership between participants and facilitators.

The distribution of medication to clients on chronic medication serves several purposes that benefit the clients as well as the health facility. Distribution of medication in the community may assist in improving access to treatment, ensure that medication is received timeously and reduce long waiting times in pharmacies at the health facilities. Clearly delivering medication in such settings is helpful, as it minimises the number of trips to the health facility and also reduces overcrowding at health facilities. This benefit could be enhanced considerably if CHWs were to advise on improving adherence and provide education relating to medication for the management of conditions. Our findings highlight the lack of adherence support given to clients with NCDs, in stark contrast to the extensive support provided by CHWs to patients on HIV infection and TB treatment.

As CHWs in this study mainly worked with individuals already known to have a chronic disease, it is crucial that they possess skills in physical rehabilitation of clients. This is particularly pertinent because of the increase in risk factors for cardiovascular disease; an increase in the aging population suggests a similar increase in the burden of cardiovascular disease in sub-Saharan Africa and elsewhere in the developing world. In the absence of appropriate interventions, stroke and heart disease-related deaths are expected to increase from 3 million in 1998 to 5 million in 2020 in developing regions as outlined by the World Health Organization.^26,27^ Such statistics suggest that there will be a need for community-based services such as CHWs with specialised skills to deal with the burden of increased numbers of people with physical disabilities caused by strokes and those who suffer from ischaemic heart disease. This will then extend services to clients in areas where there is a scarcity of rehabilitation workers or where people may have limited access to these services.

The role of CHWs in measuring vital parameters in children has been shown to be feasible (reliable) in numerous studies on children.^28^ This role could be extended to NCDs, for the detection of abnormal readings and thus the facilitation of referral. In instances when such measurements are incorrectly conducted, this may lead to CHWs’ work being devalued. Furthermore, such inadequacies in practice highlight insufficiencies in training and supervision. Thus adequate training, supervision and regular evaluation of tasks are essential in ensuring good practice.

One of the major roles for CHWs has been to connect or link community members to the health facility.^7^ In this study this role was fulfilled in numerous ways, such as the delivery of medication and referral of clients identified in the community to a health facility. In communities where health-seeking behaviour is poor and people only visit health facilities when they have serious symptoms, CHWs can play a crucial role in identifying problem cases early enough for health professionals to intervene timeously. However, it is evident that the relationship or communication between health facilities and CHWs has its challenges. Improved communication between health facilities and CHWs could assist in increasing CHWs’ legitimacy in the eyes of the community.

The role of CHWs as peer educators is an illustration of innovative thinking by their NGO in order to deal with a lack of appropriate formal training services, although innovative peer education needs to be supervised and better formalised to ensure that the knowledge transferred is appropriate and accurate. Peer education in this context shows how an organisation tailors practices to meet the needs of those they serve.

This article shows some of the limitations and challenges to the roles and responsibilities of CHWs in an NCDs programme. For example, home visits were not utilised maximally, as these could serve as places where health education is delivered. In addition, the work of CHWs concentrated on people with NCDs, thus excluding the population at risk which could benefit from their services. In a place where there is an increase in NCDs there is a need for primary prevention, and CHWs can be used in communities to identify those at risk.

### Limitations of the study

The study is limited by the methodology utilised. Although observations are an accepted qualitative research method, they have several constraints, one being that observations are context-specific. A second constraint is that study subjects may alter behaviour when observed by an outsider. Furthermore, observations were based on data collected from one NGO. It is thus important to remember that different organisations may organise work differently. Therefore the findings of this study indicate the issues relating to roles in managing NCDs rather than being generalisable.

### Recommendations

On the basis of the findings of this study, the following recommendations are made:

1. CHWs should be empowered with resources as well as forging links with non-health organisations so that they can better refer clients with non-health problems.
2. The main issue of this study is the role of CHWs relating to NCDs. On the one hand there are factors that may inhibit their roles, which may undermine their value in society; on the other hand, the varied roles add value to their work. There is therefore a need to develop the capacity of CHWs in a sustained manner and to create on-going education that is organised and relevant to the context in which they work. In addition, it will be crucial to explore the concept of peer education and how the process can be formalised and supported.
3. It is important to look at community-to-facility referral pathways, as this is important in linking clients to formal health services as well as in the continuity of care, especially for chronic conditions.

## Conclusion

This study shows the complexities of the work done by CHWs working with NCD-related conditions. Thus, understanding the actual roles of CHWs provides insights not only into the competencies required to enable them to fulfil their daily functions, but also into the type of training required to fill the present gaps.
